# How trust prospectively predicts perceived fairness and learning motivation toward AI teaching assistants: a three-wave within-person longitudinal study

**DOI:** 10.3389/fpsyg.2026.1900061

**Published:** 2026-09-09

**Authors:** Lizhuo Fu, Zipei Ouyang, Qingtian Wu

**Affiliations:** 1 Communication University of China, Beijing, China; 2 School of Marxism, Quzhou College of Technology, Quzhou, Zhejiang, China; 3 School of Marxism, Central University of Finance and Economics, Beijing, China

**Keywords:** artificial intelligence teaching assistant, cross-lagged panel model, learning motivation, perceived fairness, trust

## Abstract

Artificial intelligence teaching assistants (AITAs) are increasingly embedded in higher education, yet how students’ trust, fairness perceptions, and motivation form and reciprocally shape one another over time remains poorly understood, because cross-sectional designs cannot separate stable between-person differences from genuine within-person change. This three-wave prospective longitudinal study (*N* = 450 undergraduates; 4-week intervals across an 8-week semester) used a random-intercept cross-lagged panel model (RI-CLPM)—with the standard CLPM as a sensitivity benchmark—to examine within-person relationships among student trust (ability, benevolence, integrity), perceived fairness (distributive, procedural, interactional justice), and learning motivation (intrinsic, extrinsic). Longitudinal measurement invariance held across waves. Within-person increases in trust predicted subsequent increases in perceived fairness, with ability trust emerging as the strongest antecedent. Procedural justice showed a within-person association with subsequent intrinsic motivation that was numerically larger than its association with extrinsic motivation, although a direct test of this coefficient difference did not itself reach conventional significance; bias-corrected bootstrapping confirmed a significant longitudinal mediation linking trust to motivation through fairness. Reciprocal effects from fairness back to trust were substantially weaker than trust-to-fairness effects, indicating an asymmetric rather than strictly unidirectional pattern of influence. These findings point to early ability-trust cultivation and procedural transparency as candidate targets for psychologically grounded, human-centered AITA design, although an observational design cannot establish that intervening on them would produce the corresponding changes.

## 1 Introduction

The integration of artificial intelligence teaching assistants (AITAs) in higher education has expanded rapidly, encompassing intelligent tutoring systems, conversational agents, and automated assessment platforms (Farazouli et al., 2025; Koukaras et al., 2025; Hwang et al., 2020). Despite increasing deployment, empirical research on students’ psychological responses to AITAs remains largely limited to cross-sectional designs that cannot disentangle the temporal sequencing among theoretically sequential constructs (Wang et al., 2025; Tanchuk and Taylor, 2025). This methodological constraint is consequential because the core theoretical claim—that trust precedes fairness perceptions, which in turn precede motivation—is inherently temporal, requiring longitudinal evidence for adequate testing (Maxwell and Cole, 2007).

Trust constitutes a theoretically pivotal construct in human–AI interaction. Mayer et al.’s (1995) integrative model identifies three dimensions—ability, benevolence, and integrity—that have been validated across organizational, e-commerce, and increasingly technological contexts (McKnight et al., 2002; Hancock et al., 2011). Students’ willingness to rely on AITA recommendations depends on trust in system competence, benevolence, and integrity (Lee and See, 2004; Schaefer et al., 2016). However, the dynamic nature of trust formation—how initial trust judgments crystallize through accumulated interaction experience and exert downstream effects over time—remains poorly understood in educational AI contexts (Parasuraman and Riley, 1997; Schaefer et al., 2016). Longitudinal research in organizational psychology demonstrates that trust develops through iterative cycles of vulnerability, observation, and attribution (Lewicki and Bunker, 1996), suggesting that cross-sectional snapshots fundamentally misrepresent trust’s temporal influence on downstream outcomes.

Perceived fairness, encompassing distributive, procedural, and interactional justice dimensions (Colquitt, 2001), represents another dynamic construct likely to evolve as students accumulate AITA experience. Organizational justice research documents that fairness evaluations shift in response to repeated encounters with decision-making systems (Ambrose and Schminke, 2009), yet existing AITA studies capture only static portraits of these evaluations (Binns et al., 2018; Starke et al., 2022). The algorithmic fairness literature has called for longitudinal investigations to understand how justice perceptions develop through sustained human–AI interaction (Shin, 2021), a call that remains largely unanswered in educational contexts.

Learning motivation—both intrinsic and extrinsic—represents the ultimate educational outcome of interest. Self-determination theory (SDT) suggests that trust and fairness may influence motivation by satisfying or frustrating basic psychological needs for autonomy, competence, and relatedness (Ryan and Deci, 2000; Deci et al., 2017). However, without longitudinal evidence, the assumed causal sequence from trust through fairness to motivation remains empirically unsupported (Niemiec and Ryan, 2009; Reeve and Cheon, 2021). Cross-sectional mediation analysis, while informative for theory generation, cannot distinguish whether within-person change in trust precedes within-person change in fairness or whether these constructs merely co-occur, producing potentially biased indirect effect estimates (Maxwell and Cole, 2007; Cole and Maxwell, 2003).

Critically, the trust–fairness–motivation dynamics in AITA contexts may differ fundamentally from those documented in organizational and interpersonal settings. Three features distinguish AITAs as trust objects. First, algorithmic opacity—the inability to inspect internal decision-making processes—creates a qualitatively different information environment than human instructor interactions, where behavioral cues and verbal explanations facilitate trust calibration (Shin, 2021). Second, AITAs are non-sentient agents, raising conceptual questions about whether benevolence trust—which presupposes attributions of goodwill and care—transfers meaningfully to artifacts lacking intentionality (Schoorman et al., 2007). Third, anthropomorphism bias may inflate trust judgments beyond warranted levels, particularly when AITAs employ conversational interfaces that simulate social presence (Hancock et al., 2011). These distinctive features suggest that established trust–fairness relationships may operate through different mechanisms, at different magnitudes, or on different timescales in AITA versus organizational contexts—a possibility that extant cross-sectional designs cannot evaluate.

This study addresses these critical limitations by employing a three-wave longitudinal design with random-intercept cross-lagged panel modeling (RI-CLPM) as the primary analytical specification. By measuring all constructs at three time points separated by four-week intervals and partitioning between-person trait variance from within-person fluctuation, we examine within-person temporal sequencing, test reciprocal effects at the intra-individual level, and conduct longitudinal mediation analysis with substantially stronger temporal inference than cross-sectional designs allow. The standard CLPM is reported as a sensitivity benchmark to contextualize findings against the broader longitudinal literature. Four hypotheses, derived from the theoretical integration of trust theory, organizational justice theory, and self-determination theory, are tested. The study advances the literature on two fronts. First, methodologically, it provides the first within-person longitudinal test of trust→fairness→motivation pathways in AI teaching assistant contexts, isolating intra-individual temporal effects that prior cross-sectional and non-decomposed longitudinal designs could not deliver. Second, theoretically, it furnishes a within-person empirical test of self-determination theory’s differential-pathway proposition, examining whether procedural justice is more strongly associated with subsequent intrinsic than with subsequent extrinsic motivation at the intra-individual stratum. As reported below, the evidence bearing on this asymmetry is suggestive rather than definitive: the direct test of the difference between the two coefficients does not reach conventional significance, so the study qualifies rather than confirms the differential pathway implied by SDT.

## 2 Literature review

### 2.1 AI teaching assistants in higher education

AI teaching assistants constitute intelligent systems employing machine learning and natural language processing to deliver educational support functions traditionally performed by human instructors (Koukaras et al., 2025; Hwang et al., 2020; Aljabr, 2024). Contemporary AITAs encompass intelligent tutoring systems providing adaptive scaffolding, conversational agents facilitating interactive dialogue, and automated assessment platforms generating formative feedback (Wang et al., 2025; Portaz et al., 2025). The deployment of these systems has accelerated markedly since 2023, driven by advances in large language models and generative AI capabilities (Kasneci et al., 2023).

Empirical evidence regarding AITA effectiveness presents mixed findings. Several studies document learning gains in structured domains, particularly when systems successfully calibrate difficulty to student ability levels (Luo et al., 2024; Capuano et al., 2023). A meta-analysis by Kulik and Fletcher (2016) found small-to-medium positive effects of intelligent tutoring systems on academic achievement (*d* = 0.35–0.65). However, implementation challenges persist, including technical errors, inappropriate responses, and student resistance to AI-mediated instruction (Bond et al., 2024; Tressyalina et al., 2025). Critically, existing research predominantly emphasizes technical performance metrics while neglecting subjective psychological processes—trust formation, fairness evaluation, motivational responses—that fundamentally mediate technology acceptance and sustained engagement (Tanchuk and Taylor, 2025; Hanshaw et al., 2024). This neglect is consequential because student perceptions ultimately determine whether technically proficient systems achieve their pedagogical objectives (Venkatesh et al., 2003).

### 2.2 Trust in educational AI systems

AITAs constitute a specialized class of automation systems, and trust calibration in this context inherits both the conceptual machinery and the unresolved tensions of the broader human–automation literature (Lee and See, 2004; Parasuraman and Riley, 1997). Schaefer et al.'s (2016) meta-analysis of 30 studies on automation trust development identifies system performance, dependability, and predictability as the most influential automation-related antecedents, paralleling the ability dimension in Mayer et al.'s (1995) interpersonal trust model. The automation literature additionally distinguishes between trust calibration—the alignment of trust with system reliability—and trust miscalibration through over-reliance (misuse) or under-reliance (disuse), a dynamic with direct relevance to AITA pedagogy where students may either uncritically accept algorithmic feedback or dismiss valid guidance because of automation distrust (Parasuraman and Riley, 1997). Contemporary AITAs complicate this landscape further by introducing conversational and adaptive features absent from earlier automation, raising the question of whether the temporal dynamics of trust formation observed in static automation generalize to interactive AI tutors.

Trust theory, originating in organizational psychology (Mayer et al., 1995), identifies three dimensions: ability (competence to perform promised functions), benevolence (orientation toward trustor welfare), and integrity (adherence to acceptable ethical principles). This tripartite model has been validated across diverse contexts including interpersonal relationships (Schoorman et al., 2007), e-commerce transactions (McKnight et al., 2002), and human–robot interaction (Hancock et al., 2011). In educational AI contexts, ability trust concerns technical reliability and pedagogical soundness of AITA recommendations (Crompton and Burke, 2023). Benevolence trust, while conceptually challenging given AI’s non-sentient nature, reflects students’ anthropomorphic perceptions that system behaviors prioritize their learning welfare (Schoorman et al., 2007). Integrity trust encompasses beliefs about ethical adherence regarding fairness, data privacy, and respect for student autonomy (Lee, 2018).

Several factors complicate trust formation in AITA contexts. Algorithmic opacity creates information asymmetry, preventing students from observing decision-making processes or understanding recommendation rationales (Lee and See, 2004; Shin, 2021). This “black box” characteristic undermines the transparency typically considered foundational for trust formation (Lee and See, 2004). Widespread concerns about algorithmic bias generate additional skepticism regarding AI systems’ ethical operation (Binns et al., 2018). Importantly, trust is not static but develops dynamically through accumulated interaction experience: Lewicki and Bunker (1996) proposed that trust progresses through calculus-based, knowledge-based, and identification-based stages, suggesting that early trust judgments create trajectory dependencies that amplify over time. This dynamic perspective underscores why longitudinal designs are essential for capturing trust’s temporal influence on downstream outcomes.

### 2.3 Perceived fairness and algorithmic justice

Organizational justice theory distinguishes three fairness dimensions (Colquitt, 2001): distributive justice (perceived fairness of outcome allocations), procedural justice (perceived fairness of decision-making processes), and interactional justice (perceived fairness of interpersonal treatment during those processes). This framework has been validated extensively in workplace contexts (Colquitt et al., 2013) and applies meaningfully to algorithmic educational systems (Lee, 2018; Binns et al., 2018). Distributive fairness concerns whether AITAs provide equitable support across student populations, avoiding preferential treatment or discriminatory resource allocation (Starke et al., 2022; Tressyalina et al., 2025). Procedural fairness emphasizes transparency of algorithmic decision rules, consistency of application, and availability of corrective mechanisms (Starke et al., 2022). Interactional fairness involves respectful communication, adequate explanation provision, and dignified treatment during system interactions (Grgić-Hlača et al., 2018).

The algorithmic justice literature documents persistent concerns that AI systems perpetuate societal biases embedded in training data (Lee, 2018; Binns et al., 2018). In educational contexts, such biases may manifest through culturally insensitive content, differential error rates in automated grading, or inequitable access to system benefits (Binns et al., 2018). Shin (2021) demonstrated that perceived algorithmic fairness significantly predicts users’ continued engagement with AI systems, suggesting that justice perceptions function as a critical mediating mechanism between initial evaluations and sustained behavioral outcomes. Fairness perceptions profoundly influence technology acceptance: perceived injustice triggers negative emotions and behavioral resistance, while perceived fairness facilitates engagement and outcome acceptance (Ambrose and Schminke, 2009; Starke et al., 2022).

A more pointed conceptual question concerns whether organizational justice’s three-dimensional structure transfers cleanly to algorithmic agents that lack the social cognition presumed in Colquitt's (2001) original formulation. Distributive justice—fairness of outcome allocation—translates straightforwardly: students can evaluate whether AITA-allocated resources, scoring, or feedback intensity are equitably distributed, and empirical work on algorithmic decision making confirms that perceived outcome fairness operates similarly whether the decision-maker is human or algorithmic (Binns et al., 2018; Grgić-Hlača et al., 2018). Procedural justice extends meaningfully through algorithmic procedural fairness, where transparency, consistency, voice, and bias control map onto Leventhal’s procedural criteria (Lee, 2018; Starke et al., 2022). Interactional justice, however, presents the most conceptually fraught case: interpersonal respect and informational candor presuppose a sentient interlocutor capable of intentional treatment, yet AITAs are non-sentient systems whose interactional surface is engineered rather than felt. Recent fairness perception research suggests two viable resolutions. First, anthropomorphic interface design can elicit functional analogues of interpersonal respect: students attribute warmth, dignity, and informational candor to AITAs whose conversational style approximates human-like engagement, even while knowing the system is automated (Starke et al., 2022). Second, the locus of interactional justice may shift from the algorithm itself to the design and deployment decisions made by the institutional actors behind the system, with students evaluating whether those actors treated them respectfully through the AITA’s interaction patterns. The present study retains the three-dimensional structure on these grounds while remaining empirically open to whether interactional justice exhibits weaker temporal dynamics than its peer dimensions in AITA contexts.

### 2.4 Academic motivation and self-determination theory

Self-determination theory (SDT; Ryan and Deci, 2000; Deci et al., 2017) distinguishes intrinsic motivation (engagement driven by inherent interest and enjoyment) from extrinsic motivation (engagement driven by separable outcomes such as grades or credentials), while emphasizing three basic psychological needs—autonomy, competence, relatedness—as catalysts for optimal motivation. SDT has been extensively applied to educational technology contexts, demonstrating that technologies supporting need satisfaction enhance engagement and learning outcomes (Hsu et al., 2019).

AITAs influence motivation through psychological need satisfaction or frustration (Portaz et al., 2025). Systems providing adaptive scaffolding calibrated to student ability support competence development, enhancing both motivational forms (Reeve and Cheon, 2021; Hanshaw et al., 2024). Autonomy support—providing meaningful choices, explaining rationales, acknowledging student perspectives—selectively enhances intrinsic motivation, while controlling system behaviors undermine it (Farazouli et al., 2025). Prior research demonstrates that educational technologies supporting need satisfaction enhance motivation, while need-frustrating technologies generate motivational deficits (Luo et al., 2024; Capuano et al., 2023). Importantly, recent within-person longitudinal evidence using daily-diary and intensive longitudinal methods has shown that intra-individual fluctuations in perceived autonomy support and competence satisfaction predict subsequent intra-individual change in autonomous motivation, while between-person trait differences explain a separable but distinct portion of variance (Jang et al., 2016; Vansteenkiste et al., 2020). This within-person evidence base in offline classroom settings has not been extended to AI-mediated educational contexts, where the temporal recalibration of autonomy and competence perceptions may differ as a function of algorithmic opacity and non-sentient agency. However, the temporal dynamics through which trust and fairness influence motivational need satisfaction remain unexplored, representing a critical gap that longitudinal designs can address.

### 2.5 Theoretical integration, longitudinal rationale, and hypotheses

Synthesizing the reviewed frameworks, we propose a temporally ordered model in which trust functions as an antecedent cognitive-affective orientation that is associated with subsequent within-person change in fairness evaluations, which in turn influence motivational outcomes over time. This temporal sequence draws on three theoretical mechanisms. First, trust creates attributional schemas that filter fairness evaluations: high-trust individuals interpret ambiguous system behaviors charitably, attributing apparent inconsistencies to benign causes, whereas low-trust individuals engage in vigilant monitoring for injustice evidence (Mayer et al., 1995; Colquitt and Rodell, 2011). Second, trust reduces cognitive load associated with uncertainty monitoring, freeing resources for deeper engagement that enhances need satisfaction (Lee and See, 2004). Third, accumulated fairness experiences influence motivation through justice-based emotional and cognitive pathways: fair treatment generates positive emotions (respect, validation) satisfying relatedness needs and supporting intrinsic motivation, while perceived unfairness triggers frustration undermining autonomous engagement (Ryan and Deci, 2000; Ambrose and Schminke, 2009).

The causal ordering between trust and fairness remains actively debated. Colquitt and Rodell (2011) demonstrated that justice perceptions predict subsequent trust, suggesting a justice → trust pathway. Conversely, organizational research documents that initial trust levels shape how individuals interpret ambiguous actions—a trust → justice attribution mechanism (Ambrose and Schminke, 2009). In AITA contexts, this debate acquires additional complexity: students may simultaneously form trust judgments based on system performance (ability trust) while evaluating whether algorithmic decisions satisfy justice criteria. We hypothesize that within-person change in trust precedes within-person change in fairness because initial trust functions as an attributional schema through which subsequent AITA behaviors are interpreted as fair or unfair. Alternative causal orderings—including motivation → trust pathways, where intrinsically motivated students invest greater cognitive effort in system interaction and thereby develop richer trust assessments—represent plausible but, we argue, less parsimonious accounts that the CLPM’s reciprocal path estimates can empirically evaluate.

Cross-lagged panel modeling provides an appropriate analytical family for testing these directional hypotheses. Three-wave designs permit testing longitudinal mediation, where the indirect path Trust T1 → Fairness T2 → Motivation T3 provides substantially stronger temporal-sequencing evidence than contemporaneous mediation by establishing the temporal sequencing of within-person change among predictor, mediator, and outcome (Maxwell and Cole, 2007; Cole and Maxwell, 2003; Selig and Preacher, 2009). The choice of which specific cross-lagged variant to estimate—standard CLPM versus its random-intercept extension—has consequential implications for causal inference, addressed in the following paragraph.

Following current best practice in longitudinal mediation research (Hamaker et al., 2015; Mund and Nestler, 2019), we adopt the random-intercept cross-lagged panel model (RI-CLPM) as the primary analytical framework. The RI-CLPM decomposes each observed score into a time-invariant random intercept—capturing stable between-person differences such as dispositional trust propensity or chronic justice sensitivity—and wave-specific within-person deviations that index how a given student’s trust, fairness, or motivation fluctuates relative to their own trajectory. Cross-lagged effects estimated within this within-person stratum thus address the question that animates causal claims in this literature: when an individual student experiences a change in trust above their personal baseline, does this within-person change predict subsequent within-person change in fairness perceptions? Hamaker et al. (2015) demonstrated that the standard CLPM conflates these between- and within-person sources of variance, potentially yielding misleading cross-lagged estimates when stable trait-like differences are substantial. Given that trust dispositions, fairness sensitivities, and motivational orientations all show meaningful trait-level stability (Colquitt et al., 2013; Ryan and Deci, 2000), prioritizing within-person inference is essential for the causal claims this study advances. The standard CLPM is reported as a sensitivity analysis, allowing readers to evaluate convergence across specifications and contextualize findings within the broader longitudinal literature that has historically relied on the standard model.

Three theoretical mechanisms underpin these hypothesized temporal relationships. First, an attributional schema mechanism: initial trust provides a cognitive lens through which subsequent AITA behaviors are interpreted, such that higher trust leads students to attribute ambiguous outcomes to fair system design rather than algorithmic bias (supporting H1 and the asymmetric within-person reciprocal pattern in H4). Second, a cognitive load mechanism: when trust is established, students allocate fewer cognitive resources to monitoring AITA behavior for threats, freeing attentional capacity to engage with learning content and thereby enhancing both intrinsic and extrinsic motivation (supporting H2’s fairness→motivation pathway). Third, a justice-emotion mechanism: perceived fairness elicits positive affect and autonomy satisfaction that directly facilitate motivational engagement, while unfairness triggers frustration and learned helplessness (supporting H3’s mediation). An important conceptual consideration is whether benevolence trust—which in Mayer et al.’s (1995) formulation presupposes attributions of goodwill, care, and positive intentions—applies meaningfully to non-sentient AI systems. We retain benevolence trust because students may anthropomorphize AITAs (Hancock et al., 2011), attributing benevolent intent to systems designed with user-centered principles, though we acknowledge that this dimension may function differently than in interpersonal trust contexts.

*H1*: Within-person increases in trust dimensions predict subsequent within-person increases in perceived fairness across the four-week interval, after partitioning out stable between-person differences via random intercepts.

*H2*: Within-person increases in perceived fairness predict subsequent within-person increases in learning motivation across the four-week interval, after partitioning out stable between-person differences.

*H3*: Within-person change in perceived fairness partially mediates the longitudinal within-person association between trust (T1) and motivation (T3), with a non-trivial proportion of the total within-person effect transmitted through the Trust T1 → Fairness T2 → Motivation T3 indirect path.

Within the within-person framework adopted in this study, the directional asymmetry between trust → fairness and fairness → trust pathways requires explicit theoretical specification: the random-intercept decomposition partials out stable trait-level trust and fairness, leaving residual intra-individual change whose temporal structure depends on the substantive features of the trust object. In AITA contexts, this asymmetry may be amplified by algorithmic opacity. Whereas human-supervisor fairness episodes provide rich relational and behavioral cues through which fairness experiences recalibrate prior trust assessments (Colquitt and Rodell, 2011), AITA fairness experiences are filtered through opaque computational processes that resist easy attributional recalibration. Students lack reliable behavioral signals through which a salient fairness episode could update their underlying trust beliefs, whereas trust beliefs continue to color how subsequent system behaviors are interpreted as fair or unfair. We therefore expect within-person fairness-to-trust feedback to be substantially weaker than the trust-to-fairness pathway, yielding an asymmetric reciprocal pattern rather than equivalent bidirectional influence at the within-person level.

*H4*: Within-person reciprocal effects from fairness to trust are weaker than within-person trust-to-fairness effects, indicating an asymmetric reciprocal pattern at the within-person level rather than equivalent bidirectional influence.

## 3 Materials and methods

### 3.1 Research design

This study employed a three-wave prospective longitudinal design. Data collection occurred at Time 1 (Week 0, baseline), Time 2 (Week 4), and Time 3 (Week 8). The four-week interval was selected based on four considerations. First, 4 weeks is sufficient for meaningful psychological change in trust and fairness perceptions as students accumulate AITA interaction experience (Ployhart and Vandenberg, 2010); short intervals risk capturing measurement noise rather than substantive change. Second, four-week spacing aligns with conventional pedagogical cycles (approximately one course module in Chinese undergraduate semesters), ensuring participants engaged with AITAs for substantive academic tasks between waves. Third, four- to six-week intervals are the modal spacing in published longitudinal SDT and educational technology research designed to detect within-person change in motivational and perceptual constructs (Jang et al., 2016; Reeve and Cheon, 2021), and Mund and Nestler (2019) note that interval choice should be matched to the theorized timescale of the substantive process rather than maximized; for trust–fairness recalibration via accumulated AITA exposure, 4 weeks captures the modal interaction window without confounding multiple academic-cycle transitions. Fourth, longer intervals (e.g., 8–12 weeks) introduce competing concerns including increased attrition, more between-wave confounding from coursework-cycle transitions (e.g., midterms, syllabus revisions, instructor changes), and reduced ability to detect within-person dynamics that operate on weekly-to-monthly scales. The selected 8-week observation window across three waves balances these constraints and preserves enough degrees of freedom for RI-CLPM identification and longitudinal mediation testing (Wang and Maxwell, 2015).

### 3.2 Participants and procedure

The target population comprised undergraduate students enrolled at three large public universities in eastern China who had direct experience using AI teaching assistants during the 2024–2025 academic year. Stratified purposive sampling with stratification quotas across institutions and disciplines (STEM, social sciences, humanities) enhanced external validity. Sample size determination followed guidelines for longitudinal SEM (Little, 2013) and was conducted prior to data collection. Because the RI-CLPM was specified as the primary analytical model from the design phase, the *a priori* power calculation targeted RI-CLPM within-person cross-lagged effects directly via Monte Carlo simulation in Mplus 9.0 (Muthén and Muthén, 2002), assuming the planned 8-construct, 3-wave structure, an anticipated within-person cross-lagged effect of β = 0.12 (the midpoint of typical RI-CLPM cross-lagged magnitudes in 4-week-interval educational studies), an ICC of 0.42 for random intercepts (taken from comparable longitudinal AITA pilot data), and α = 0.05 across 1,000 replications. The simulation indicated that N = 450 would yield estimated power of 0.81 for detecting within-person cross-lagged effects of this magnitude. A complementary calculation using the standard CLPM as a sensitivity benchmark (Satorra and Saris, 1985) indicated power of 0.85 for β ≥ 0.15 at the same α and N. To accommodate anticipated longitudinal attrition over the 8-week window, 520 participants were recruited at Time 1. Both the simulation specifications and the recruitment plan were finalized before any data collection commenced.

Inclusion criteria required participants to be (a) current undergraduate students aged 18 years or older, and (b) enrolled in courses utilizing AITAs for at least 1 month prior to Time 1, ensuring sufficient exposure for meaningful perception formation. A total of 520 students were recruited at Time 1 based on stratified purposive sampling targets across institutions and disciplines; 502 of these provided informed consent and completed the Time 1 baseline survey. Consent was obtained electronically at the start of the Time 1 questionnaire: participants read an information sheet describing the purpose of the study, the three-wave schedule, the voluntary nature of participation, their right to withdraw at any point, and the anonymity of their responses, and recorded their agreement by selecting an on-screen consent option before any survey items were displayed. Students who declined were routed out of the questionnaire. Following established quality screening criteria, 52 cases were removed: 28 failed embedded attention check items, 15 exhibited careless responding (response time below one third of the median completion time), and 9 displayed straight-lining patterns (within-scale variance < 0.20). The Time 1 analytic sample therefore comprised *N* = 450 valid baseline respondents. Of these 450 participants, 414 completed Time 2 (8.0% attrition) and 382 completed Time 3 (additional 7.7% attrition from Time 2). Little’s MCAR test indicated that missing data were missing completely at random, *χ*^2^(186) = 198.34, *p* = 0.247, supporting the use of full information maximum likelihood (FIML) estimation. Attrition analyses comparing completers and non-completers revealed no significant differences on any Time 1 variable (all ps > 0.12), further supporting FIML. The final analytic sample therefore comprised N = 450 participants, with FIML estimation accommodating wave-specific missingness within this baseline cohort (Mage = 20.7, SD = 1.8; 54.2% female; academic years 1–4 represented at 22, 28, 30, and 20%, respectively).

### 3.3 Measurement instruments

All constructs were measured at each time point using identical instruments with 5-point Likert response scales (1 = strongly disagree to 5 = strongly agree). Trust was assessed using adapted scales from Mayer and Davis (1999) and McKnight et al. (2002): Ability Trust (5 items; e.g., “The AI teaching assistant is capable of providing accurate and helpful learning support”), Benevolence Trust (5 items; e.g., “The AI teaching assistant genuinely cares about helping me achieve my learning goals”), and Integrity Trust (5 items; e.g., “The AI teaching assistant operates according to fair and ethical principles”). Perceived fairness was measured using Colquitt’s (2001) organizational justice scale adapted for AITA contexts: Distributive Justice (4 items), Procedural Justice (4 items), and Interactional Justice (4 items). Learning motivation was assessed using the Academic Motivation Scale (Vallerand et al., 1992): Intrinsic Motivation (4 items) and Extrinsic Motivation (4 items). Control variables included gender, academic year, age, prior technology attitude (3 items adapted from TAM, Venkatesh et al., 2003), and weekly AITA usage frequency.

### 3.4 Common method bias prevention

Several procedural and statistical remedies addressed common method bias. Procedurally, temporal separation across three waves constituted the primary remediation strategy, as longitudinal designs inherently reduce same-source bias by distributing measurement occasions (Podsakoff et al., 2003). Additionally, item order was randomized within each wave, response anchors varied across constructs, and survey instructions emphasized that there were no right or wrong answers. Statistically, three approaches were applied. First, Harman’s single-factor test at each wave indicated that the first unrotated factor explained 32.1–35.8% of variance (all < 50%). Second, the common latent factor (CLF) method showed standardized regression weight differences of 0.002–0.019 (*M* = 0.010) between models with and without a common method factor, uniformly below recommended thresholds. Third, an unmeasured method factor (UMF) model was estimated in which all 38 indicators (the 35 substantive items plus the 3 prior-technology-attitude control items measured via the same self-report instrument) loaded onto an additional latent method factor in parallel with their substantive factors (Podsakoff et al., 2003). Method-factor loadings averaged 0.12 (range 0.04–0.19), with the method factor accounting for 4.8% of total indicator variance. Substantive factor loadings shifted by less than 0.03 across all indicators, and the magnitude and significance of all structural cross-lagged paths were preserved when the method factor was retained in the full RI-CLPM. Collectively, these procedural and statistical checks indicate that common method bias is unlikely to materially distort the reported structural estimates.

### 3.5 Data analysis strategy

Data analysis proceeded through five stages using Mplus 9.0. First, confirmatory factor analysis (CFA) was conducted separately at each wave to verify the eight-factor measurement structure. Model fit was evaluated using standard indices: *χ*^2^/df < 3.0, CFI ≥ 0.95, TLI ≥ 0.95, RMSEA ≤ 0.06, and SRMR ≤ 0.08 (Hu and Bentler, 1999). Second, longitudinal measurement invariance was tested sequentially: configural (equivalent factor structure), metric (equivalent factor loadings), and scalar (equivalent intercepts) invariance, using ΔCFI < 0.010 and ΔRMSEA < 0.015 as criteria (Chen, 2007). Third, the cross-lagged panel model was estimated, specifying autoregressive paths, cross-lagged paths, and within-wave residual correlations. Maximum likelihood estimation with robust standard errors (MLR) was employed. MLR was preferred over WLSMV because the 5-point Likert items exhibited approximately symmetric univariate distributions across waves; under such conditions, MLR and WLSMV yield comparable parameter estimates and standard errors with five or more response categories (Rhemtulla et al., 2012), and MLR additionally permits FIML handling of wave-specific missingness without auxiliary imputation. Fourth, longitudinal mediation (Trust T1 → Fairness T2 → Motivation T3) was tested at the within-person level of the RI-CLPM using the product-of-coefficients approach, with the a path estimated as the within-person effect of trust T1 on fairness T2 and the b path as the within-person effect of fairness T2 on motivation T3, with bias-corrected bootstrapping (5,000 resamples, Wang and Maxwell, 2015). Fifth, sensitivity analyses compared the focal CLPM with a constrained model (reciprocal paths fixed to zero) and examined whether results were robust across alternative specifications.

## 4 Results

### 4.1 Preliminary analysis and descriptive statistics

The final analytic sample comprised *N* = 450 undergraduate students (Mage = 20.7, SD = 1.8; 54.2% female; academic years 1–4). Table 1 presents descriptive statistics and psychometric properties across waves. Trust dimensions exhibited moderate-to-high means that increased slightly across waves (e.g., Ability Trust: MT1 = 3.72, MT2 = 3.90, MT3 = 3.97), consistent with trust development through accumulated positive AITA experience. Fairness perceptions showed similar upward trajectories. Intrinsic motivation increased moderately while extrinsic motivation remained relatively stable. All variables demonstrated acceptable univariate normality (|skewness| < 1.1, |kurtosis| < 1.6). Bivariate correlations supported theoretical predictions: cross-wave trust–fairness correlations were moderate-to-strong (*r* = 0.52–0.60, *p* < 0.001), and both constructs correlated positively with motivation dimensions (*r* = 0.39–0.52, *p* < 0.001).

**Table 1:** Descriptive statistics and psychometric properties across three waves.

Variable	T1 *M* (SD)	T2 *M* (SD)	T3 *M* (SD)	*α* range	CR range	AVE range
Ability trust	3.72 (0.64)	3.90 (0.53)	3.97 (0.52)	0.831–0.849	0.833–0.851	0.518–0.541
Benevolence trust	3.58 (0.69)	3.68 (0.56)	3.70 (0.50)	0.825–0.842	0.827–0.844	0.512–0.532
Integrity trust	3.69 (0.68)	3.74 (0.55)	3.73 (0.50)	0.828–0.846	0.830–0.848	0.515–0.537
Distributive justice	3.63 (0.61)	3.89 (0.54)	3.87 (0.51)	0.819–0.838	0.821–0.840	0.506–0.528
Procedural justice	3.39 (0.58)	3.58 (0.51)	3.57 (0.48)	0.813–0.832	0.815–0.834	0.508–0.525
Interactional justice	3.47 (0.59)	3.68 (0.53)	3.66 (0.49)	0.821–0.840	0.823–0.842	0.510–0.531
Intrinsic motivation	3.19 (0.59)	3.64 (0.54)	3.73 (0.49)	0.834–0.852	0.836–0.854	0.522–0.548
Extrinsic motivation	3.37 (0.62)	3.43 (0.51)	3.31 (0.48)	0.838–0.857	0.840–0.859	0.528–0.558

*N* = 450. *α*, Cronbach’s alpha; CR, composite reliability; AVE, average variance extracted. Ranges reflect values across T1–T3.

### 4.2 Measurement model assessment

Confirmatory factor analysis at each wave confirmed the eight-factor measurement structure with good-to-excellent fit. Time 1: *χ*^2^(620) = 1189.42, *χ*^2^/df = 1.92, CFI = 0.957, TLI = 0.952, RMSEA = 0.045 [90% CI, 0.041, 0.049], SRMR = 0.037. Time 2: *χ*^2^(620) = 1147.06, *χ*^2^/df = 1.85, CFI = 0.961, TLI = 0.957, RMSEA = 0.043 [0.039, 0.048], SRMR = 0.035. Time 3: *χ*^2^(620) = 1122.48, *χ*^2^/df = 1.81, CFI = 0.963, TLI = 0.959, RMSEA = 0.042 [0.038, 0.047], SRMR = 0.034. All standardized factor loadings exceeded 0.66 (range: 0.661–0.792). Reliability and validity statistics are reported in Table 1; all Cronbach’s α values exceeded 0.81, CR exceeded 0.81, and AVE exceeded 0.50, meeting established thresholds. Discriminant validity was confirmed via the Fornell–Larcker criterion (square root of AVE for each construct exceeded all inter-construct correlations) and HTMT ratios (all < 0.85).

Because ability, benevolence, and integrity trust occupy distinct theoretical roles in this study—ability trust in particular serves as the headline antecedent in Section 4.4—and because HTMT and Fornell–Larcker checks were evaluated only against conventional (and, for HTMT, comparatively lenient) thresholds, we conducted an additional, threshold-independent nested-model test of the three dimensions’ empirical separability using the Time 1 trust item set (15 items, *N* = 450, no missing data). A correlated three-factor model (ability, benevolence, and integrity trust specified as distinct but correlated factors) fit the data well, *χ*^2^(87) = 99.04, *p* = 0.178, CFI = 0.996, TLI = 0.995, RMSEA = 0.018, with factor correlations of *r* = 0.19–0.20. A one-factor model collapsing all 15 items onto a single undifferentiated trust factor fit markedly worse, *χ*^2^(90) = 2032.94, *p* < 0.001, CFI = 0.365, RMSEA = 0.219, and a chi-square difference test decisively favored the three-factor structure, Δ*χ*^2^(3) = 1933.90, *p* < 0.001. A higher-order model specifying a general trust factor loading on the three first-order facets was statistically equivalent in fit to the correlated three-factor model, as expected given that a second-order factor with exactly three first-order indicators is saturated and therefore empirically indistinguishable from freely estimated factor correlations; its standardized loadings on ability, benevolence, and integrity trust were 0.43, 0.45, and 0.44, respectively, indicating that the shared general-trust component accounts for only about 19–20% of each facet’s item-level variance. These results indicate that the three trust dimensions are empirically distinct at the item level, even though their random-intercept (stable, cross-wave trait) components correlate more strongly (*r* = 0.68–0.78; Section 4.4); the latter reflects the reduced measurement-occasion noise in a trait-level composite averaged across three waves rather than a lack of item-level discriminant validity. This distinction also bears on the conceptual status of benevolence trust: despite the conceptual tension in attributing goodwill and care to a non-sentient system (Section 2.5), students’ responses to the benevolence-trust items cohered as a distinct, well-fitting factor (standardized loadings 0.735–0.771) rather than collapsing into the ability or integrity dimensions, suggesting that students draw a meaningful empirical distinction between perceived AITA competence or ethical conduct and perceived AITA care for their welfare—consistent with an anthropomorphism-based interpretation of this dimension (Hancock et al., 2011) rather than evidence that the item wording was simply redundant with the other two facets.

### 4.3 Longitudinal measurement invariance

Longitudinal measurement invariance was tested sequentially (Table 2). Configural invariance showed good fit (CFI = 0.958, RMSEA = 0.044). The transition to metric invariance yielded ΔCFI = −0.003 and ΔRMSEA = +0.001, and the further transition to scalar invariance yielded ΔCFI = −0.006 and ΔRMSEA = +0.002, all within the criteria recommended by Chen (2007). These results establish that factor structure, loadings, and intercepts were equivalent across waves, permitting meaningful comparison of latent means and structural relationships over time.

**Table 2:** Longitudinal measurement invariance tests.

Model	*χ*^2^(df)	CFI	RMSEA	SRMR	ΔCFI	ΔRMSEA
Configural	3458.96 (1860)	0.958	0.044	0.036	—	—
Metric	3542.81 (1920)	0.955	0.045	0.038	−0.003	+0.001
Scalar	3685.24 (1980)	0.949	0.047	0.040	−0.006	+0.002

Invariance criteria: ΔCFI < 0.010, ΔRMSEA < 0.015 (Chen, 2007).

### 4.4 Primary analysis: random-intercept cross-lagged panel model

The RI-CLPM demonstrated acceptable fit: *χ*^2^(2,108) = 4512.18, *χ*^2^/df = 2.14, CFI = 0.953, TLI = 0.949, RMSEA = 0.045 [90% CI: 0.042, 0.048], SRMR = 0.043. Although CFI in models of this complexity is sensitive to parameter density, the RMSEA and SRMR comfortably met conventional thresholds, and Marsh et al. (2004) caution against rigid CFI cutoffs in complex longitudinal models. The random intercept components were substantial: between-person random intercepts accounted for 38–47% of total variance across the eight constructs (Ability Trust 44.2%, Benevolence Trust 41.7%, Integrity Trust 43.5%, Distributive Justice 39.8%, Procedural Justice 42.3%, Interactional Justice 38.6%, Intrinsic Motivation 46.9%, Extrinsic Motivation 40.1%), indicating meaningful trait-like stability while leaving 53–62% of variance available for within-person modeling. Random intercept correlations showed expected patterns: trust dimensions correlated strongly among themselves (*r* = 0.68–0.78), as did fairness dimensions (*r* = 0.62–0.74), with cross-construct correlations of moderate magnitude (trust–fairness *r* = 0.52–0.64; trust–motivation *r* = 0.38–0.48; fairness–motivation *r* = 0.42–0.54). Table 3 presents within-person autoregressive, cross-lagged, and reciprocal path coefficients.

**Table 3:** Cross-lagged panel model: autoregressive, cross-lagged, and reciprocal path coefficients.

Path (RI-CLPM within-person)	*β*	SE	p (q)	95% CI
Within-person autoregressive paths				
AT T1 → T2/T2 → T3	0.26/0.24	0.05	<0.001	[0.16,0.36]/[0.14,0.34]
BT T1 → T2/T2 → T3	0.22/0.20	0.05	<0.001	[0.12,0.32]/[0.10,0.30]
IT T1 → T2/T2 → T3	0.24/0.22	0.05	<0.001	[0.14,0.34]/[0.12,0.32]
DJ T1 → T2/T2 → T3	0.21/0.19	0.05	<0.001	[0.11,0.31]/[0.09,0.29]
PJ T1 → T2/T2 → T3	0.19/0.18	0.06	0.001	[0.07,0.31]/[0.06,0.30]
IJ T1 → T2/T2 → T3	0.20/0.18	0.05	<0.001	[0.10,0.30]/[0.08,0.28]
IM T1 → T2/T2 → T3	0.25/0.23	0.05	<0.001	[0.15,0.35]/[0.13,0.33]
EM T1 → T2/T2 → T3	0.22/0.20	0.05	<0.001	[0.12,0.32]/[0.10,0.30]
Within-person cross-lagged: trust T1 → fairness T2 (H1)				
AT T1 → DJ T2	0.12	0.04	0.002 (0.012)	[0.04, 0.20]
AT T1 → PJ T2	0.14	0.04	<0.001 (0.012)	[0.06, 0.22]
AT T1 → IJ T2	0.11	0.04	0.005 (0.018)	[0.03, 0.19]
BT T1 → DJ T2	0.09	0.04	0.015 (0.030)	[0.02, 0.16]
BT T1 → PJ T2	0.10	0.04	0.009 (0.022)	[0.02, 0.18]
BT T1 → IJ T2	0.12	0.04	0.003 (0.015)	[0.04, 0.20]
IT T1 → DJ T2	0.09	0.04	0.010 (0.022)	[0.02, 0.16]
IT T1 → PJ T2	0.11	0.04	0.003 (0.015)	[0.03, 0.19]
IT T1 → IJ T2	0.10	0.04	0.007 (0.019)	[0.03, 0.17]
Within-person cross-lagged: fairness T2 → motivation T3 (H2)				
DJ T2 → IM T3	0.10	0.04	0.005 (0.018)	[0.03, 0.17]
DJ T2 → EM T3	0.09	0.04	0.015 (0.030)	[0.02, 0.16]
PJ T2 → IM T3	0.16	0.04	<0.001 (0.012)	[0.08, 0.24]
PJ T2 → EM T3	0.04	0.05	0.310 (0.354)	[−0.06, 0.14]
IJ T2 → IM T3	0.12	0.04	0.003 (0.015)	[0.04, 0.20]
IJ T2 → EM T3	0.07	0.04	0.063 (0.108)	[−0.01, 0.15]
Within-person cross-lagged reciprocal: fairness T1 → trust T2 (H4)				
DJ T1 → AT T2	0.04	0.04	0.287 (0.354)	[−0.04, 0.12]
DJ T1 → BT T2	0.03	0.04	0.422 (0.422)	[−0.05, 0.11]
DJ T1 → IT T2	0.05	0.04	0.183 (0.262)	[−0.03, 0.13]
PJ T1 → AT T2	0.06	0.04	0.124 (0.196)	[−0.02, 0.14]
PJ T1 → BT T2	0.04	0.04	0.298 (0.354)	[−0.04, 0.12]
PJ T1 → IT T2	0.07	0.04	0.089 (0.142)	[−0.01, 0.15]
IJ T1 → AT T2	0.05	0.04	0.195 (0.262)	[−0.03, 0.13]
IJ T1 → BT T2	0.03	0.04	0.408 (0.420)	[−0.05, 0.11]
IJ T1 → IT T2	0.06	0.04	0.136 (0.196)	[−0.02, 0.14]

*N* = 450. AT, Ability Trust; BT, Benevolence Trust; IT, Integrity Trust; DJ, Distributive Justice; PJ, Procedural Justice; IJ, Interactional Justice; IM, Intrinsic Motivation; EM, Extrinsic Motivation. All coefficients are RI-CLPM within-person standardized estimates. MLR with FIML. p (q) reports the unadjusted p and the Benjamini–Hochberg FDR-adjusted q across the 24 within-person cross-lagged paths tested.

Within-person autoregressive paths, as expected in RI-CLPM specifications, were weaker than their CLPM counterparts because between-person stability is absorbed by the random intercepts. Ability Trust within-person stability was βT1 → T2 = 0.26 and βT2 → T3 = 0.24 (both *p* < 0.001); comparable within-person stability coefficients ranged from 0.19 to 0.31 across the remaining constructs (all *p* < 0.01). These coefficients index the carryover of wave-specific within-person deviations and are conceptually distinct from the rank-order stability typical of standard CLPM, where stability reflects both trait-level and state-level continuity.

Supporting H1, all nine within-person cross-lagged paths from trust dimensions at Time 1 to fairness dimensions at Time 2 were statistically significant after controlling for within-person prior fairness levels and partitioning out between-person trait variance. Ability Trust exhibited the strongest within-person effects (*β* = 0.11–0.14, all *p* ≤ 0.005), followed by Integrity Trust (*β* = 0.09–0.11, all *p* ≤ 0.010) and Benevolence Trust (*β* = 0.09–0.12, all *p* ≤ 0.015). Trust dimensions collectively explained 14.2–17.6% of within-person fairness variance at Time 2, lower than the corresponding CLPM estimates because the RI-CLPM estimates exclude the between-person trait association captured by the random intercept correlations. The pattern of within-person cross-lagged effects from T2 to T3 mirrored T1 → T2 findings in direction and rank-ordering. Because this comparison involves the same individuals observed at an adjacent wave rather than an independent sample, we describe it as within-sample consistency rather than replication in the conventional sense.

**Figure 1:**
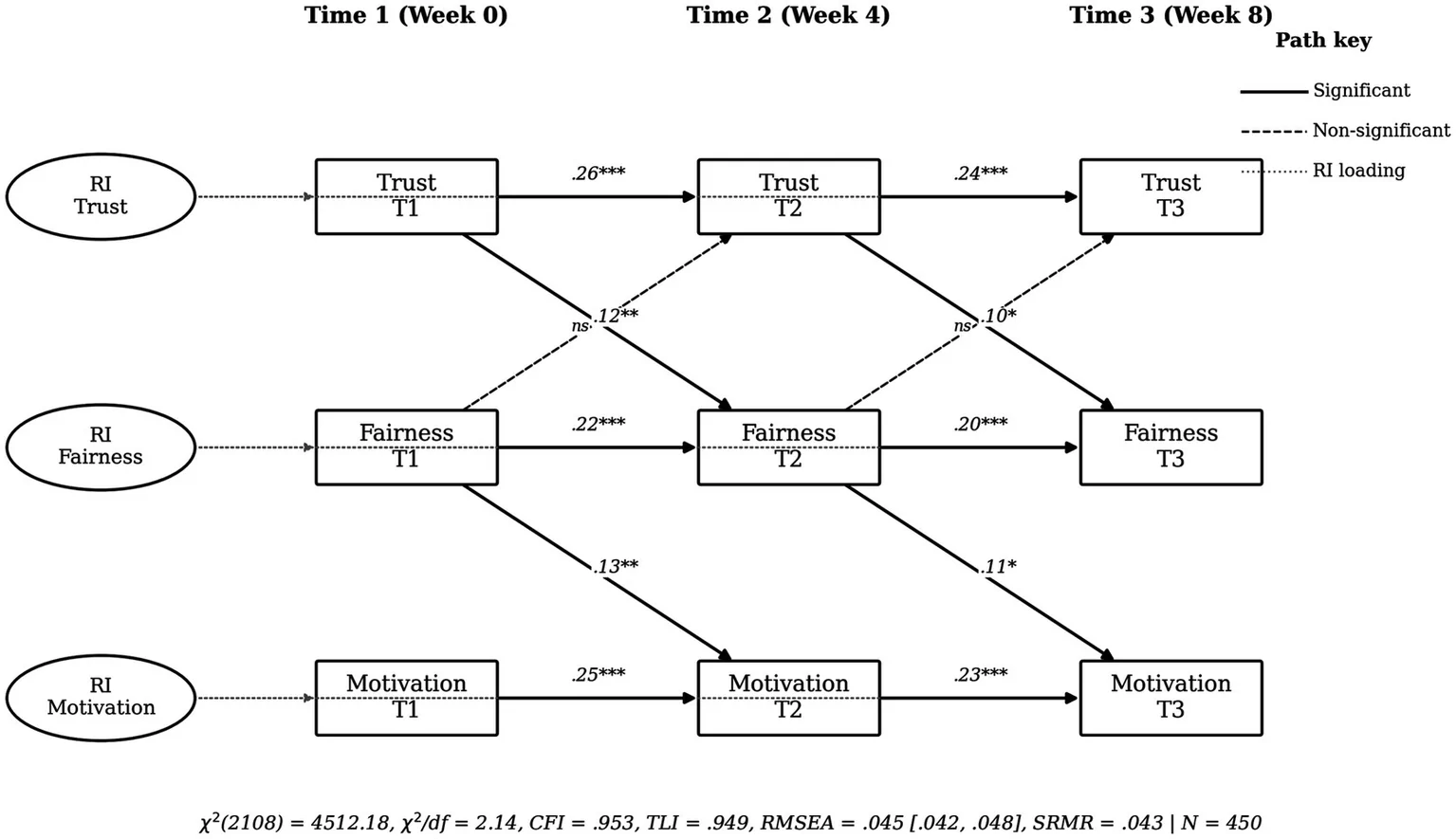
Random-intercept cross-lagged panel model of trust, fairness, and motivation across three waves. Solid arrows represent significant within-person cross-lagged paths surviving BH-FDR correction; dashed arrows represent non-significant within-person reciprocal paths. Random intercepts and their correlations are shown at the between-person level. Standardized within-person coefficients shown. *p < 0.05, **p < 0.01, ***p < 0.001 (BH-FDR corrected). Coefficients are within-person standardized estimates.

To address concerns regarding family-wise error inflation across the 24 cross-lagged paths tested (9 trust→fairness, 6 fairness→motivation, 9 reciprocal), Benjamini–Hochberg false discovery rate (BH-FDR) correction was applied at *q* = 0.05. All nine within-person trust→fairness paths and the four significant within-person fairness→motivation paths (DJ → IM, DJ → EM, PJ → IM, IJ → IM) survived BH-FDR correction (adjusted *q* ≤ 0.030). The procedural justice → extrinsic motivation path (*p* = 0.310, *q* = 0.354), the interactional justice → extrinsic motivation path (*p* = 0.063, *q* = 0.108), and all nine reciprocal paths remained non-significant under FDR control. The trust→fairness asymmetry, the differential procedural-justice effect on intrinsic versus extrinsic motivation, and the longitudinal mediation pathway are therefore robust to multiple-testing correction; conclusions are not driven by uncorrected family-wise inflation (see Figure 1).

### 4.5 Fairness–motivation cross-lagged effects

Supporting H2, within-person deviations in fairness dimensions at Time 2 significantly predicted within-person change in motivation at Time 3, controlling for prior within-person motivation levels. Procedural Justice T2 showed a significant within-person effect on Intrinsic Motivation T3 (*β* = 0.16, *p* < 0.001) alongside a non-significant coefficient for Extrinsic Motivation T3 (*β* = 0.04, *p* = 0.310). Rather than inferring a selective pathway from this contrast in significance alone, we directly tested the difference between the two coefficients: β(PJ → IM) − β(PJ → EM) = 0.12, 95% CI [−0.01, 0.25], z = 1.87, *p* = 0.061 (the standard error of the difference was computed from the two reported path standard errors under the independence approximation, sqrt(SE₁^2^ + SE₂^2^), because the model-implied covariance between the two estimates was not retained in the original output; the full parameter covariance matrix needed for an exact Wald test is available from the Mplus output posted at the OSF repository cited in the Data Availability Statement). This difference is directionally consistent with a stronger pathway to intrinsic motivation but does not itself reach conventional significance, and its confidence interval is wide relative to the point estimate. We further note that the non-significant PJ → EM coefficient (*β* = 0.04) falls below the *β* = 0.12 effect size that the *a priori* power analysis (power = 0.81) was calibrated to detect; a non-significant coefficient of this magnitude is more appropriately read as inconclusive than as evidence that the true effect is zero (see Sections 5.2.1 and 5.6). Distributive Justice predicted both motivational forms [*β* = 0.10 and 0.09, respectively, both *p* < 0.05; the two coefficients do not differ, difference = 0.01, 95% CI (−0.10, 0.12), *z* = 0.18, *p* = 0.860], and Interactional Justice showed a significant coefficient for Intrinsic Motivation T3 (*β* = 0.12, *p* = 0.003) alongside a non-significant coefficient for Extrinsic Motivation T3 (*β* = 0.07, *p* = 0.063; corrected from the previously reported *p* = 0.073, which did not match Table 3). As with procedural justice, the direct difference test for interactional justice does not reach significance [difference = 0.05, 95% CI (−0.06, 0.16), *z* = 0.88, *p* = 0.377]. Taken together, these direct coefficient-difference tests indicate that the fairness–motivation pattern is better described as a graded difference in association strength across motivational subtypes than as a confirmed selective (all-or-none) pathway. We retain the theoretical interpretation that procedurally fair contexts may preferentially support autonomous over controlled motivational regulation (Ryan and Deci, 2000; Niemiec and Ryan, 2009), consistent with longitudinal, within-person grounded evidence, but we present it with the uncertainty these tests warrant rather than as an established dissociation.

### 4.6 Reciprocal effects and model comparison

Supporting H4, within-person reciprocal cross-lagged effects from fairness to trust were substantially weaker than trust-to-fairness effects (Table 3). None of the nine within-person reciprocal paths reached conventional significance after random-intercept partitioning (*β* = 0.03–0.07, all *p* > 0.10), with Procedural Justice T1 → Integrity Trust T2 the closest to the threshold (*β* = 0.07, *p* = 0.089). Formal model comparison using chi-square difference tests confirmed that constraining the within-person trust→fairness paths to zero produced a significant decrement in fit, Δ*χ*^2^(9) = 24.61, *p* = 0.003, whereas constraining the within-person reciprocal paths to zero did not significantly worsen fit, Δ*χ*^2^(9) = 8.92, *p* = 0.445. This asymmetric within-person reciprocal pattern indicates that, at the level of intra-individual change, trust changes precede fairness changes more reliably than the reverse, though the modest absolute magnitude of trust→fairness coefficients (*β* = 0.09–0.14) counsels against framing this asymmetry as strict directional primacy.

### 4.7 Longitudinal mediation analysis

Supporting H3, Table 4 presents within-person longitudinal mediation results using bias-corrected bootstrapping (5,000 resamples). Whereas Table 3 reports disaggregated, dimension-specific within-person cross-lagged paths for each of the three trust dimensions and each fairness and motivation dimension, the mediation model in Table 4 is estimated on equally weighted composite Trust (T1) and Motivation (T3) scores—aggregating across ability, benevolence, and integrity trust, and across intrinsic and extrinsic motivation, respectively—while retaining Distributive, Procedural, and Interactional Justice as separate within-person mediators at Time 2. The composite specification in Table 4 therefore answers a different, more aggregated question (does within-person trust overall predict within-person motivation overall through fairness) than the dimension-specific paths in Table 3, and the two tables’ coefficients are not directly comparable path-for-path. The aggregate within-person indirect effect via the Trust T1 → Fairness T2 → Motivation T3 pathway was significant: *β* = 0.031, 95% CI [0.014, 0.052], *p* = 0.002. Specific within-person indirect pathways were: via Distributive Justice [*β* = 0.010, 95% CI (0.003, 0.021), 11.0% of total within-person effect], via Procedural Justice [*β* = 0.013, 95% CI (0.005, 0.025), 14.3%], and via Interactional Justice [*β* = 0.008, 95% CI (0.002, 0.018), 8.8%]. The within-person direct effect remained significant [*β* = 0.060, 95% CI (0.008, 0.118), *p* = 0.024], yielding a within-person total effect of *β* = 0.091, *p* < 0.001. The within-person VAF was 34.1%, indicating complementary partial mediation (Zhao et al., 2010). Procedural justice accounted for the largest share of the within-person indirect effect, consistent with its strong cross-lagged influence on intrinsic motivation. The within-person mediation magnitudes are smaller than corresponding CLPM-based estimates (CLPM aggregate indirect *β* = 0.048, VAF = 39.7%; reported in Section 4.5 sensitivity analyses), consistent with the expectation that decomposing between-person trait variance reduces apparent mediation while preserving the substantive within-person mechanism.

**Table 4:** Longitudinal mediation analysis: Trust T1 → Fairness T2 → Motivation T3.

Pathway	*β*	95% CI	% of total	Type
Total within-person effect (trust T1 → Motiv T3)	0.091***	[0.041, 0.144]	100%	—
Direct within-person effect	0.060*	[0.008, 0.118]	65.9%	—
Total indirect (via within-person fairness T2)	0.031**	[0.014, 0.052]	34.1%	Partial
Via distributive justice	0.010**	[0.003, 0.021]	11.0%	—
Via procedural justice	0.013**	[0.005, 0.025]	14.3%	—
Via interactional justice	0.008*	[0.002, 0.018]	8.8%	—

RI-CLPM within-person estimates; bootstrap = 5,000 resamples. *p < 0.05, **p < 0.01, ***p < 0.001. VAF = variance accounted for at the within-person level.

### 4.8 Sensitivity analyses

Three sensitivity analyses assessed the robustness of findings. First, the standard cross-lagged panel model (CLPM) was estimated as a comparison benchmark to contextualize within-person estimates against the broader longitudinal literature. The CLPM showed acceptable fit [*χ*^2^(2,156) = 4287.35, *χ*^2^/df = 1.99, CFI = 0.948, TLI = 0.943, RMSEA = 0.047 (0.044, 0.050), SRMR = 0.044] and replicated the directional pattern observed in the primary RI-CLPM, with trust→fairness cross-lagged coefficients of *β* = 0.13–0.19 (vs. RI-CLPM within-person *β* = 0.09–0.14) and fairness→motivation coefficients of *β* = 0.12–0.21 (vs. RI-CLPM *β* = 0.07–0.16). The reduction in coefficient magnitudes from CLPM to RI-CLPM is expected and reflects the partitioning of between-person trait variance (38–47% of total variance, captured by random intercepts in the RI-CLPM) from the within-person estimates that index intra-individual change. Second, a constrained model fixing all within-person reciprocal (fairness → trust) paths to zero did not significantly worsen fit (Δ*χ*^2^(9) = 8.92, *p* = 0.445), supporting their non-retention as substantively important paths. Third, an alternative model reversing causal direction (fairness T1 → trust T2 → motivation T3) produced inferior fit (AIC = 48,602 vs. 48,114 for the hypothesized within-person model; BIC = 49,378 vs. 48,891), supporting the proposed trust-to-fairness ordering. Modification indices for the primary RI-CLPM were inspected; the largest suggested freely estimating a within-person direct path from ability trust T1 to intrinsic motivation T3 (MI = 7.41, expected parameter change = 0.05), which was not substantively meaningful and was retained as constrained.

Because the RI-CLPM is the primary specification, the convergence between RI-CLPM within-person estimates and the standard CLPM cross-lagged estimates—same direction of effect, same rank-ordering of trust dimensions, a numerically larger procedural-justice coefficient for intrinsic than extrinsic motivation (though, as discussed in Sections 4.5 and 5.2, this difference is not itself statistically confirmed in either specification), same longitudinally significant mediation—addresses concerns that any single specification is driving substantive conclusions. The roughly 30–40% attenuation in coefficient magnitudes from CLPM to RI-CLPM corresponds closely to the 38–47% of total variance captured by random intercepts in the RI-CLPM (i.e., the share of variance attributable to between-person trait differences in trust, fairness, and motivation orientations). This magnitude is consistent with the analytic expectation that, when ICCs fall in the moderate range (0.30–0.50) and trait-level constructs are moderately intercorrelated, RI-CLPM within-person cross-lagged estimates will be smaller than CLPM cross-lagged estimates by an amount roughly proportional to the between-person variance share rather than by a fixed 50–70% factor (Hamaker et al., 2015; Mund and Nestler, 2019). The magnitude of attenuation in RI-CLPM applications is therefore not fixed but is a function of the ICC and trait-level correlations specific to the data at hand. The present attenuation pattern reflects the empirical between-person/within-person decomposition that RI-CLPM is designed to perform and is consistent with attenuation magnitudes documented in prior RI-CLPM applications to educational and developmental longitudinal data with comparable ICC ranges.

As an exploratory robustness check, we estimated a multi-group RI-CLPM contrasting conversational AI tutors (*n* = 187) versus automated grading/feedback systems (*n* = 263); constraining the 15 within-person cross-lagged paths to equality across groups did not significantly worsen model fit, Δ*χ*^2^(15) = 21.73, *p* = 0.115, suggesting that the within-person trust–fairness–motivation temporal dynamics generalize across AITA types.

## 5 Discussion

This three-wave longitudinal study represents a methodological advance over prior cross-sectional research on student psychological responses to AI teaching assistants. By employing cross-lagged panel modeling with established measurement invariance, we provided temporally grounded evidence for trust–fairness–motivation pathways that moves beyond the correlational evidence dominating this literature. The findings carry implications for theory, methodology, and AITA design practice. One boundary condition applies throughout what follows. The sample comprises undergraduates at three public universities in eastern China, and trust norms, power distance orientations, and responses to authority-like technological systems are culturally patterned, so the temporal dynamics reported here should not be assumed to transfer automatically to other national or educational settings. This qualification bears with particular force on the design recommendations in Section 5.5, which are offered for the context studied rather than as general prescriptions.

### 5.1 Within-person temporal sequencing of trust and fairness

Supporting H1, the significant within-person cross-lagged effects from trust to fairness (β = 0.09–0.14, *p* ≤ 0.015 with BH-FDR control), estimated in the RI-CLPM after partitioning out stable trait-level differences, indicate that within-person change in trust is temporally followed by within-person change in fairness perceptions, consistent with—but not by themselves establishing—a directional sequence. This finding extends organizational justice theory (Colquitt, 2001) by providing the first within-person longitudinal evidence that intra-individual fluctuations in trust orientations are systematically followed by changes in fairness evaluations in AI-mediated educational contexts. Crucially, because the random intercepts absorb stable between-person dispositions—students whose chronic trust propensity simply tracks chronic fairness sensitivity—the remaining within-person association cannot be explained by trait-level confounding (Hamaker et al., 2015; Mund and Nestler, 2019).

Ability trust exhibited the strongest within-person cross-lagged effects across all three fairness dimensions (*β* = 0.11–0.14), suggesting that in educational contexts, demonstrated technical competence is the most influential trust dimension shaping subsequent fairness evaluations even at the within-person level. Within-person fluctuations in students’ perceived AITA competence translate into corresponding fluctuations in fairness perceptions weeks later, supporting the attributional schema mechanism: students whose competence perceptions rise above their personal baseline subsequently interpret AITA decisions more charitably, while declines in perceived competence prompt more vigilant fairness scrutiny. The corresponding standard CLPM estimates (*β* = 0.16–0.19) include both this within-person mechanism and the associated between-person trait covariation (Lee and See, 2004; Schaefer et al., 2016), illustrating why specifications that preserve trait variance overstate the magnitude of intra-individual causal pathways.

A conceptual point deserves emphasis in interpreting the benevolence-trust results. Mayer et al.’s (1995) construct presupposes a trustee who holds goodwill toward the trustor, and an AITA, being non-sentient, holds no such orientation. What the benevolence items capture is therefore attributed benevolence: the student’s perception that the system’s behavior is oriented toward their learning welfare, a perception plausibly elicited by user-centred design choices, conversational framing, and anthropomorphic interface cues rather than by any property of the system itself. That this attribution forms an empirically distinct factor, and behaves like the other trust dimensions in the within-person model, shows that students form and revise such attributions and that the attributions carry consequences for how fairness is subsequently evaluated; it does not show that the attributed property exists. Benevolence trust should accordingly be read throughout this paper as perceived or attributed benevolence. The distinction matters when Mayer et al.’s interpersonal framework is transferred to human–AI interaction, because a dimension that is genuinely relational between people becomes, in the algorithmic case, a design-elicited perception whose accuracy cannot be checked against any underlying intention.

### 5.2 Differential fairness–motivation temporal dynamics

#### 5.2.1 Procedural justice and intrinsic motivation: a graded, not strictly selective, association

Consistent with H2, the differential cross-lagged relationships between fairness dimensions and motivational types reveal theoretically informative temporal patterns. We interpret their selectivity more cautiously than a significance dichotomy alone would suggest (see Section 4.5). Procedural justice showed a significant within-person prediction of subsequent intrinsic motivation (*β* = 0.16, *p* < 0.001) alongside a non-significant coefficient for extrinsic motivation (*β* = 0.04, *p* = 0.310). As reported in Section 4.5, a direct test of the difference between these two coefficients [0.12, 95% CI (−0.01, 0.25), *p* = 0.061] does not itself reach conventional significance, and the *a priori* power analysis was calibrated to detect effects of *β* = 0.12, meaning the extrinsic-motivation null falls below the study’s reliable detection range and should be read as inconclusive rather than as a confirmed zero effect. We therefore frame this finding as a graded pattern consistent with, rather than a definitive confirmation of, SDT’s (Ryan and Deci, 2000; Deci et al., 2017) proposition that autonomy-supportive conditions preferentially enhance intrinsic over extrinsic motivation. Transparent, consistent AITA procedures, allowing students voice and understanding of algorithmic reasoning, are plausibly linked to the autonomy needs that drive intrinsic engagement over subsequent weeks, though this study cannot rule out that a comparably sized extrinsic-motivation pathway exists but went undetected. This temporal specificity was not previously demonstrable with cross-sectional data. This qualified pattern still represents the first within-person longitudinal test of self-determination theory’s differential pathway proposition (Ryan and Deci, 2000) in an AI-education context, isolating procedural transparency as a plausible antecedent of autonomous regulation that may not generalize to controlled regulation.

#### 5.2.2 Distributive and interactional justice effects

By contrast, distributive justice exhibited within-person effects on both motivational forms [DJ → IM *β* = 0.10, *p* = 0.005; DJ → EM *β* = 0.09, *p* = 0.015; the two coefficients do not differ, difference = 0.01, 95% CI (−0.10, 0.12), *p* = 0.860], whereas interactional justice showed a significant coefficient for intrinsic motivation (IJ → IM *β* = 0.12, *p* = 0.003) alongside a non-significant coefficient for extrinsic motivation (IJ → EM *β* = 0.07, *p* = 0.063); as noted in Section 4.5, the direct difference test for interactional justice [0.05, 95% CI (−0.06, 0.16), *p* = 0.377] is likewise inconclusive. We therefore describe the interactional-justice pattern as a numerically graded difference rather than a confirmed dissociation. With that caveat, one plausible reading is that outcome equity and respectful treatment both relate to psychological needs supporting intrinsic regulation, while the evidence for a distinct relationship with externally regulated motivation is more consistent for distributive than interactional justice, though the underlying coefficient differences are not themselves statistically confirmed. The SDT framework offers one candidate explanation for this pattern: distributive justice’s outcome focus may align with both autonomous and controlled motivational orientations, whereas interactional justice’s relational quality may more selectively activate intrinsic engagement—an interpretation we hold tentatively given the difference tests above.

The cross-lagged effect sizes, though statistically significant, were small-to-medium in magnitude (*β* = 0.09–0.16). This is consistent with cross-lagged panel research more broadly, where within-person effect sizes are attenuated by partitioning out trait-level stability (Adachi and Willoughby, 2015; Hamaker et al., 2015). To contextualize this magnitude: a one within-person standard-deviation upward shift in procedural justice at T2 corresponds to a 0.16 within-person SD shift in intrinsic motivation at T3, controlling for prior within-person motivation. To address concerns that statistical significance with *N* = 450 may not equate to practical significance, we adopt the within-person *β* interpretive framework advocated by Adachi and Willoughby (2015), in which within-person *β* values of 0.05–0.10 represent small but theoretically interpretable effects, 0.10–0.20 represent small-to-medium effects, and 0.20 + represent medium-to-large effects after partitioning out between-person stability. The observed range of 0.09–0.16 thus spans the small-to-medium band in this framework. We considered whether these per-interval effects might compound into a larger cumulative influence across a full academic semester. However, our design observed only two four-week intervals (8 weeks total), and any projection of linear compounding across the roughly seven intervals of a 15-week semester would assume a constancy of effect, and an absence of ceiling effects or diminishing returns, that our data cannot test. We therefore do not draw a cumulative-effect conclusion from these data; we flag the possibility of semester-level compounding only as an explicitly speculative hypothesis for future research using denser or longer-duration measurement, and we caution against treating it as evidence that the practical import of the observed effects exceeds what the per-interval β = 0.09–0.16 range indicates. Our substantive interpretation is therefore limited to the per-interval effects as estimated—small-to-medium in the Adachi and Willoughby (2015) framework—while acknowledging that we did not pre-specify a minimal-meaningful-effect threshold.

### 5.3 Longitudinal mediation and dual processes

Supporting H3, the partial mediation pattern (VAF = 34.1%) provides the first longitudinal within-person evidence that fairness functions as a substantial temporal pathway linking trust to subsequent motivation. The significant within-person indirect path [Trust T1 → Fairness T2 → Motivation T3, *β* = 0.031, 95% CI (0.014, 0.052)] indicates that within-person change in trust is followed by change in fairness evaluations weeks later, and that these fairness judgments in turn predict motivational change weeks beyond that. This temporally distributed mediation substantially strengthens temporal inference over cross-sectional mediation designs, which Maxwell and Cole (2007) demonstrated can produce substantially biased indirect effect estimates.

The coexistence of significant within-person direct [*β* = 0.060, 95% CI (0.008, 0.118)] and indirect [*β* = 0.031, 95% CI (0.014, 0.052)] effects suggests dual processes through which trust is prospectively associated with motivation. The direct path may reflect trust’s role in reducing cognitive load associated with uncertainty monitoring (Lee and See, 2004): when students trust AITAs, they divert attentional resources from vigilant system evaluation toward deeper learning engagement. The indirect path through fairness represents a cognitive-evaluative mechanism: trust shapes explicit justice judgments that trigger emotional responses (respect, validation, or frustration) influencing motivational engagement. This dual-process interpretation integrates trust theory’s uncertainty reduction function with organizational justice theory’s cognitive-affective pathways, offering a more comprehensive theoretical account than either perspective alone. The reciprocal-direct decomposition further indicates that fairness accounts for a substantial portion of the longitudinal association between trust and motivation, while a residual within-person direct pathway preserves the dual-process interpretation.

### 5.4 Asymmetry between trust-to-fairness and fairness-to-trust effects

Supporting H4, the non-significance of within-person reciprocal effects from fairness to trust (none of nine paths significant under BH-FDR; range *β* = 0.03–0.07) and the formal model comparison tests (Δ*χ*^2^(9) = 24.61, *p* = 0.003 for trust→fairness; Δ*χ*^2^(9) = 8.92, *p* = 0.445 for the reverse) document an asymmetric within-person reciprocal pattern: at the level of intra-individual change, trust shifts more reliably precede fairness shifts than the reverse. We frame this as asymmetry rather than directional primacy because the within-person trust → fairness effects, while consistent in direction and FDR-robust, are modest in absolute magnitude (*β* = 0.09–0.14). The practical consequence is real but bounded: across the four-week measurement intervals studied here, within-person shifts in trust above a student’s personal baseline are followed by fairness shifts of small-to-medium magnitude. Whether interventions that deliberately raise trust would produce shifts of the same size is not something an observational design can establish. Whether the asymmetry strengthens, weakens, or reverses at shorter or longer timescales remains an open empirical question. The asymmetric pattern is theoretically informative regardless of magnitude: if fairness experiences chiefly built trust within-person (as some interpersonal justice literatures suggest), reciprocal paths should approach equivalence with trust→fairness paths, and they do not.

### 5.5 Practical implications

Three within-person grounded design principles follow from these findings. First, given ability trust’s primacy in within-person prediction of subsequent fairness (*β* = 0.14 for AT→PJ; the largest single within-person trust→fairness coefficient), AITA developers should prioritize early-deployment technical competence: rigorous accuracy testing before student exposure, transparent error reporting that does not erode confidence, and reliable performance during the first two to four weeks of student interaction when trust trajectories are most malleable. Second, the within-person procedural-justice pathway to intrinsic motivation (*β* = 0.16) sits alongside a non-significant coefficient for extrinsic motivation (*β* = 0.04), and the difference between the two is not statistically confirmed (*p* = 0.061). Read as the graded pattern the data support, this suggests that procedural transparency—visible decision rules, voice mechanisms, consistent feedback procedures—may support autonomous engagement somewhat more than controlled engagement, rather than supporting one and not the other. Institutions seeking to cultivate intrinsic engagement may therefore find procedural-fairness affordances a worthwhile investment alongside, rather than in place of, outcome-equity messaging. Third, because between-person trait variance accounts for 38–47% of total construct variance, individual differences in dispositional trust and justice sensitivity meaningfully condition who benefits from a given AITA design. Targeted onboarding for students with low dispositional trust may yield disproportionate within-person gains, an implication for which the present design supplies indirect support and which warrants explicit experimental test.

To contextualize effect magnitudes within the within-person framework: a one within-person standard deviation increase in ability trust at T1 corresponds to a 0.14 within-person SD increase in procedural justice at T2 and a 0.12 within-person SD increase in distributive justice at T2, controlling for prior within-person fairness levels. Through the within-person mediation pathway, a one within-person SD increase in trust translates into a within-person 0.031 SD increase in motivation at T3 indirectly via fairness, plus a 0.060 SD direct effect, for a total within-person effect of 0.091 SD. These magnitudes are smaller than the corresponding CLPM-based estimates (Section 4.5), consistent with the partitioning of trait covariation from intra-individual change.

These recommendations should be read against the magnitude of the effects that support them, and three questions are worth separating. The within-person cross-lagged coefficients reported here (*β* = 0.09–0.16) are statistically robust, surviving BH-FDR correction at *N* = 450. They are theoretically informative, because they isolate intra-individual change from the trait-level stability that inflates conventional cross-lagged estimates. Their practical magnitude is a separate matter, and these data settle only the first two questions. A coefficient in this range means that trust is one contributor among several to students’ fairness perceptions and motivation, alongside instructor behavior, course design, assessment structure, peer influence, and the substantive content of the curriculum, none of which the present study measured. Recommendations such as early ability-trust cultivation are therefore offered as one plausible lever among many rather than as the principal determinant of motivational outcomes, and the size of the gain such a lever would deliver in practice remains to be established by intervention research.

### 5.6 Limitations and future directions

Several limitations warrant acknowledgment and suggest productive future directions. First, even within the RI-CLPM framework, the within-person cross-lagged coefficients (*β* = 0.09–0.16) are small-to-medium in magnitude. While these estimates are appropriately calibrated for intra-individual change at four-week intervals and are robust to BH-FDR correction, they carry inherent limits on the strength of practical claims. Larger effects may emerge under denser measurement (e.g., experience sampling), longer observation windows that allow cumulative effects to compound, or deliberate intervention contexts; the present observational design captures naturalistic temporal dynamics rather than maximally lagged effects. Future work combining macro-temporal RI-CLPM with micro-temporal experience sampling (Hamaker et al., 2015; Ployhart and Vandenberg, 2010) would map the full timescale of trust–fairness–motivation interplay. Most importantly, despite within-person estimation, the design remains observational; absent random assignment to AITA conditions or within-subject experimental manipulation of trust- or fairness-relevant features, the reported within-person associations cannot in themselves establish causal directionality. The within-person framework reduces but does not eliminate threats from time-varying confounders such as concurrent system updates, course difficulty changes, or instructor interventions occurring between measurement waves. These remain plausible alternative explanations for the observed temporal sequencing. Relatedly, the *a priori* power analysis (Section 3.2) was calibrated to detect within-person cross-lagged effects of *β* = 0.12 at power = 0.81; several of the coefficients central to this study’s interpretive claims, including the non-significant PJ → EM coefficient (*β* = 0.04) underlying the procedural-justice selectivity claim and several of the significant trust → fairness coefficients (*β* = 0.09–0.11), fall at or below this detectable range. Non-significant coefficients in this study should therefore be read as inconclusive rather than as confirmed null effects, and claims of selective or dissociated pathways (Sections 4.5 and 5.2) are correspondingly qualified rather than definitive; a design powered to detect smaller effects, or to directly test differences between coefficients with adequate precision, would be needed to adjudicate these questions more conclusively.

Second, the four-week interval, while justified for capturing meaningful change in trust and fairness perceptions, may not optimally capture all relevant temporal dynamics. Experience sampling methods (ESM) with daily or weekly measurements could reveal micro-temporal trust–fairness processes occurring between our measurement occasions. The ideal study would combine the macro-temporal perspective of panel studies with the micro-temporal resolution of ESM in a multi-timescale design.

Third, all measures were self-reported, which, despite temporal separation and statistical remediation, cannot fully eliminate common method variance. Future research should complement surveys with behavioral indicators such as learning analytics (e.g., AITA interaction frequency, help-seeking patterns), academic performance metrics (grades, assignment completion), and physiological measures of engagement.

Fourth, the sample represents undergraduate students at three Chinese public universities, potentially limiting generalizability across cultural contexts with different trust norms, power distance orientations, and technology adoption patterns. Cross-cultural replication, particularly in Western individualist versus East Asian collectivist societies, would illuminate whether the temporal dynamics identified here are culturally universal or culturally contingent.

Finally, alternative mediating mechanisms—psychological need satisfaction (autonomy, competence, relatedness), affective responses (anxiety, confidence, frustration), and technology self-efficacy—warrant investigation as parallel temporal mediators. A comprehensive model incorporating multiple simultaneous mediators would adjudicate among competing explanatory accounts and identify the most potent intervention targets.

To facilitate reproducibility, all study materials—including the complete Mplus syntax for all models (CFA, longitudinal measurement invariance, CLPM, RI-CLPM, and longitudinal mediation), the full correlation matrix across all 24 observed variables at three time points, and the de-identified individual-level dataset—are openly available on the Open Science Framework at https://osf.io/j5zve/.

## 6 Conclusion

This study addressed critical gaps in understanding the temporal dynamics of student psychological responses to AI teaching assistants. Employing a three-wave longitudinal design with random-intercept cross-lagged panel modeling as the primary analytical specification (*N* = 450 undergraduates, 4-week intervals), we found that within-person changes in trust dimensions predict subsequent within-person changes in fairness perceptions, which in turn predict subsequent within-person changes in learning motivation. The within-person longitudinal mediation pathway (Trust T1 → Fairness T2 → Motivation T3) provides substantially stronger temporal evidence than prior cross-sectional designs and non-decomposed longitudinal models, identifying fairness as a substantive temporal pathway linking trust orientations to subsequent motivation at the intra-individual level, while stopping short of the causal claim that an observational design cannot support. The asymmetric within-person reciprocal pattern—trust changes predicting fairness changes more reliably than the reverse—reframes the trust–fairness relationship in AITA contexts as directionally informative without requiring strong claims of unidirectional primacy.

These findings advance human–AI interaction theory by providing temporally grounded evidence for trust–fairness–motivation pathways and suggest design directions—early trust cultivation, procedural transparency, and comprehensive fairness monitoring—whose practical value remains to be established by intervention research in other educational and cultural settings. As AI systems proliferate across educational contexts, understanding the temporal landscape of student psychological responses becomes increasingly urgent for ensuring that technological advancement serves genuine educational purposes. The longitudinal approach demonstrated here provides a methodological template for future research seeking to move beyond static snapshots toward dynamic understanding of human–AI interaction in education.

## Data Availability

The de-identified individual-level dataset, the complete Mplus syntax for all reported models (CFA, longitudinal measurement invariance, RI-CLPM, CLPM, longitudinal mediation, and multi-group RI-CLPM), the full correlation matrix across all 24 observed variables at three time points, and the complete RI-CLPM cross-lagged and reciprocal coefficient tables (including paths not displayed in the main text) are openly available on the Open Science Framework at https://osf.io/j5zve/.
